# Treatment of Severe Adenovirus Infection With HAdV‐B21 in a Young Obese Male With Normal Immunity

**DOI:** 10.1002/ccr3.71109

**Published:** 2025-10-22

**Authors:** Ji Peng, Zhen Wang, Shili Zhong

**Affiliations:** ^1^ Chongqing Red Cross Hospital Chongqing PR China; ^2^ Department of Intensive Care Medicine Army Medical Center of PLA Chongqing PR China

**Keywords:** HAdV‐B21, hormonal anti‐inflammatory therapy, immunocompetent adult, mechanical ventilation, ribavirin, severe adenovirus infection

## Abstract

Adenovirus infections are usually mild, but rare serotypes like “HAdV‐B21” can cause severe pneumonia even in healthy individuals. We report a 32‐year‐old immunocompetent obese male who developed life‐threatening HAdV‐B21 pneumonia (PaO_2_/FiO_2_ 62 mmHg), requiring mechanical ventilation. Successful treatment included ribavirin, corticosteroids, and prone positioning, avoiding ECMO. This case demonstrates the potential severity of rare adenovirus strains and supports combined antiviral/anti‐inflammatory therapy for such infections.

**Summary**

- Severe HAdV‐B21 pneumonia in a healthy obese male, successfully treated with ribavirin, steroids, and prone positioning, avoiding ECMO.
- Highlights rare adenovirus virulence and combined therapy efficacy.

## 1 Introduction

Adenoviruses constitute a diverse group of pathogens capable of causing a wide array of human diseases, ranging from mild respiratory illnesses to severe, life‐threatening conditions. These non‐enveloped, double‐stranded DNA viruses are known for their stability in the external environment, facilitating transmission and causing outbreaks in communal settings such as military barracks and healthcare facilities [1]. In immunocompetent individuals, adenovirus infections typically manifest as self‐limiting conditions affecting the respiratory tract, gastrointestinal system, or conjunctiva. However, severe forms of the disease, including pneumonia, can occur, particularly in immunocompromised patients or in the context of novel or re‐emergent adenovirus serotypes. The rarity of severe adenovirus infections in individuals with normal immunity makes each case significantly contribute to our understanding of the virus's pathogenic potential and clinical management [2].

The epidemiology of adenovirus infections reveals a complex landscape of more than 50 serotypes, each associated with different clinical presentations and varying degrees of severity [3]. Human Adenovirus B21 (HAdV‐B21) is particularly interesting due to its uncommon occurrence and the lack of data regarding its epidemiology and clinical impact. HAdV‐B21 has been sporadically reported in the literature, with cases often presenting severe symptoms and a higher risk of progression to acute respiratory distress syndrome (ARDS). This serotype's ability to cause severe disease in otherwise healthy adults highlights a critical gap in our understanding of adenovirus pathogenesis and the need for enhanced surveillance, diagnosis, and treatment strategies. Existing literature predominantly focuses on common serotypes, such as types 3 and 7, leaving a knowledge void around the clinical management of rarer serotypes like HAdV‐B21 [4].

Current challenges in treating adenovirus infections underscore the necessity for novel therapeutic approaches and a better understanding of the virus's behavior in immunocompetent hosts. The lack of specific antiviral treatments approved for adenovirus infections necessitates a reliance on supportive care and broad‐spectrum antivirals, which may not be optimally effective against all serotypes. The case of severe HAdV‐B21 infection described in this report addresses this gap by illustrating the successful management of a rare and severe adenovirus infection in an immunocompetent adult. Through this case, we explore the efficacy of combined hormonal anti‐inflammatory and ribavirin antiviral therapy, shedding light on potential treatment avenues for similar future cases. This contribution is poised to enrich the existing knowledge on adenovirus infections, providing valuable insights into the epidemiology, clinical presentation, and management of rare serotypes like HAdV‐B21 in the general population.

## 2 Case History/Examination

A 32‐year‐old yellow Asian race male with a body weight of 140 kg and no known underlying immune deficiencies presented with a 15‐day history of intermittent cough and sputum production without apparent cause. Four days before hospital admission, the patient experienced a worsening of symptoms, characterized by repeated fever peaks. In response, he self‐administered amoxicillin and anaxine without notable improvement. Subsequently, he sought treatment at a local clinic, where he received an unspecified antibiotic infusion. Persisting symptoms prompted a visit to a local health center, where a chest X‐ray was performed, though details remain unspecified. Due to deteriorating respiratory function, the patient was administered high‐flow oxygen inhalation (F 60 L/min, FiO_2_ 100%) and commenced using moxifloxacin and imipenem for anti‐infection at a local hospital. Despite these measures, the patient's condition did not significantly improve, necessitating endotracheal intubation the same afternoon. Arterial blood gas analysis post‐intubation revealed pH 7.26, PCO_2_ 66.0 mmHg, PO_2_ 62.0 mmHg, K+ 4.30 mmol/L, Na+ 134 mmol/L, and Lac 1.0 mmol/L. Laboratory tests indicated PCT 0.15 ng/mL, IL‐6 59.91 pg/mL, and normal white blood cell count. Staining and microscopy for acid‐fast bacilli, Pneumocystis carinii, nucleic acid tests for influenza A and B viruses, and novel coronavirus returned negative results.

**FIGURE 1:**
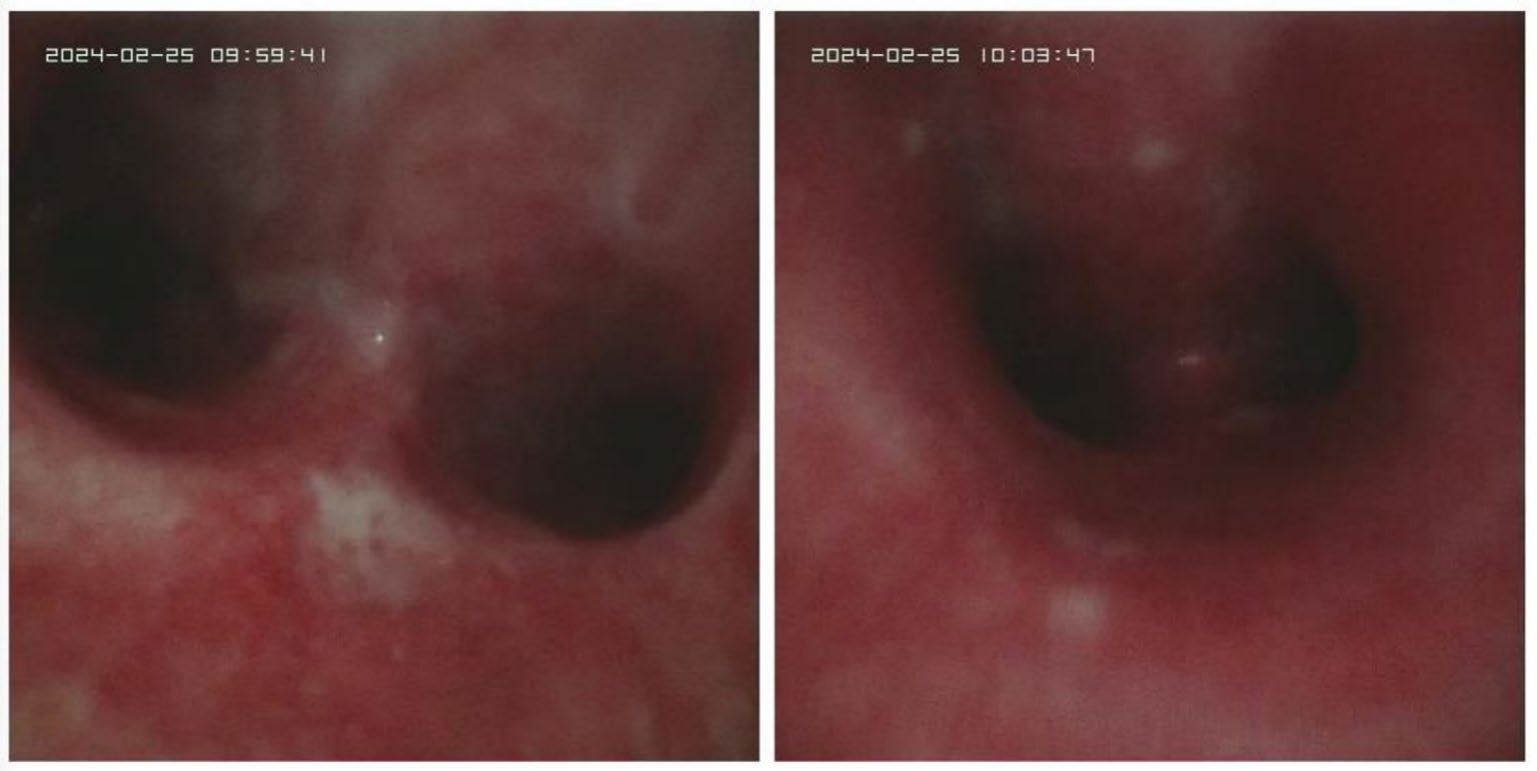
Airway condition under fiberbronchoscopy on the first day of admission.

Upon transfer to our facility due to insufficient oxygenation, the patient's oxygenation index was approximately 60 mmHg, indicative of severe pneumonia, type II respiratory failure, and severe ARDS, with an APACHE II score of 21, correlating to a mortality risk of 75.68%. The patient's family declined ECMO treatment due to financial constraints. Bronchoscopic alveolar lavage revealed bilateral airway mucosal hyperemia and edema without evidence of sputum obstruction. Following lavage (Figure 1), cultures and smears were obtained for further analysis. Treatment was initiated with azithromycin and moxifloxacin for anti‐infection, and an intravenous injection of methylprednisolone 60 mg was administered for its anti‐inflammatory effects, alongside prone ventilation, low tidal volume ventilation, plateau pressure limitation, fluid rebalance, and lung re‐expansion strategies.

**FIGURE 2:**
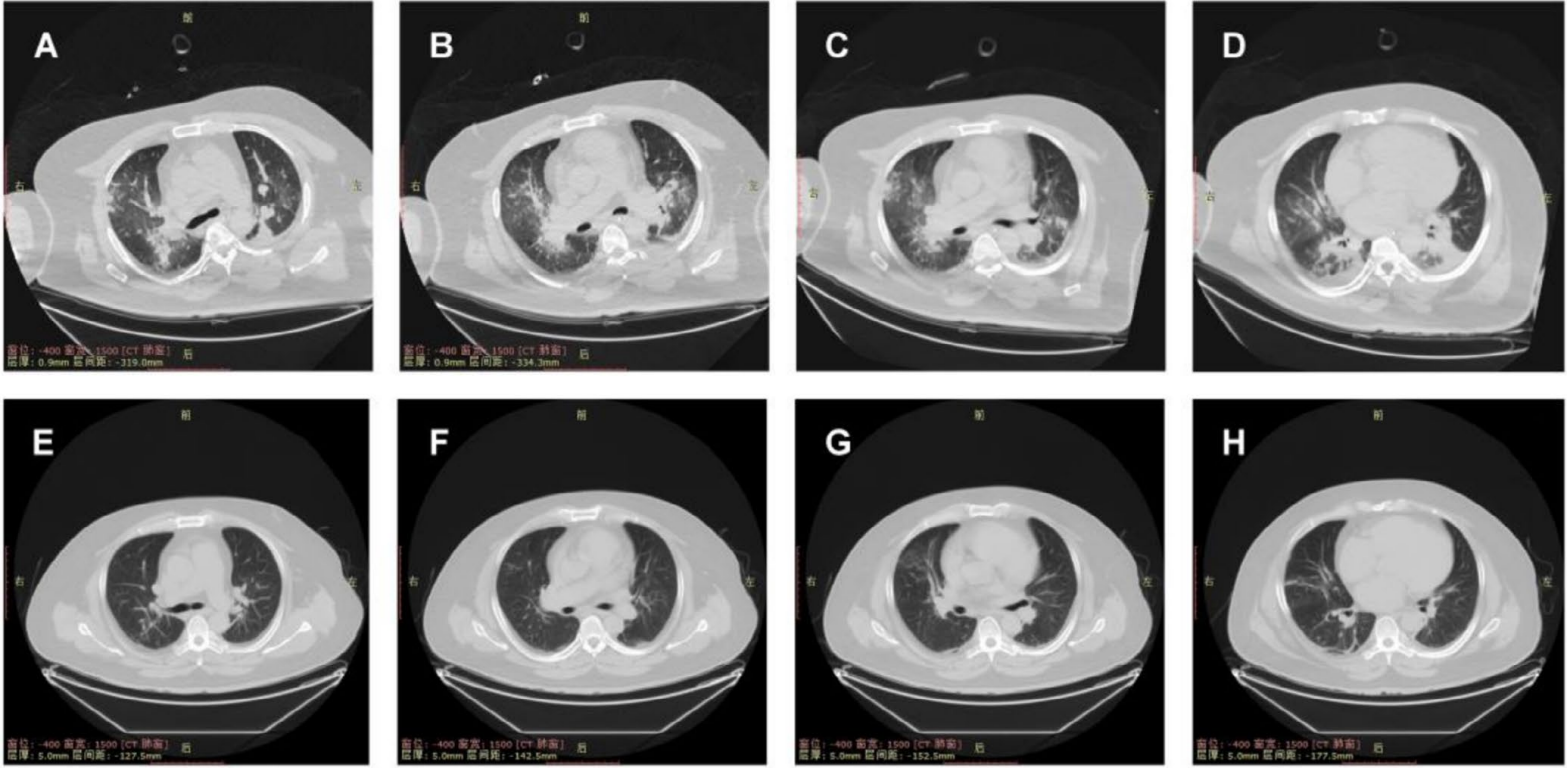
Chest CT on day 1 (A–D) and day 9 of admission (E–H).

On the second day post‐admission, the oxygen concentration was reduced to 50%, with an improved oxygenation index of approximately 120 mmHg. A subsequent chest CT scan (Figure 2A–D) and adenovirus nucleic acid test confirmed a strong positive result for adenovirus with a high titer. Ribavirin therapy was initiated at a dosage of 0.5 g every 12 h. A follow‐up chest CT on day 9 of treatment showed significant improvement (Figure 2E–H). The methylprednisolone dosage was tapered to 30 mg intravenously, and by day 10, the patient's oxygenation index had stabilized around 300 mmHg, allowing for successful ventilator weaning and tracheal intubation catheter removal.

## 3 Differential Diagnosis, Investigations and Treatment

Adult adenovirus pneumonia is very rare in clinical treatment and needs to be differentiated from influenza A and influenza B viruses. Adenovirus nucleic acid test confirmed a strong positive result for adenovirus with a high titer. The treatment regimen incorporated a comprehensive strategy involving ribavirin, corticosteroid therapy, prone positioning ventilation, lung‐protective ventilation (low tidal volume with plateau pressure restriction), negative fluid balance maintenance, and lung recruitment maneuvers.

## 4 Conclusion and Results (Outcome and Follow‐Up)

By day 13, adenovirus nucleic acid tests returned negative, marking the patient's recovery and subsequent discharge. This case emphasizes the effectiveness of a multidisciplinary treatment approach, including using ribavirin and corticosteroids, in managing severe adenovirus infection, specifically HAdV‐B21, in an immunocompetent adult. The intervention strategy, underscored by timely antiviral and anti‐inflammatory therapies, contributed significantly to the patient's recovery, underscoring the potential for such regimens in similar cases. The patient was followed up for half a year. There were no complications and the patient worked and lived normally.

## 5 Discussion

Adenoviruses constitute a significant etiology of viral pneumonia, contributing to approximately 2% of all community‐acquired pneumonia cases [5, 6]. While predominantly observed in pediatric populations, adenovirus pneumonia can also affect healthy adults, particularly those residing or working in confined or densely populated settings, such as military environments [6]. The precise mechanisms underlying the susceptibility of immunocompetent adults to adenovirus infection remain elusive. However, it is hypothesized that environmental factors, such as suboptimal sanitary conditions observed at the patient's workplace—a construction site—may play a contributory role in the pathogenesis [7]. Notably, adenoviruses exhibit resilience against a wide array of environmental disinfectants, yet their viability can be effectively neutralized by solutions containing 95% ethanol.

In cases of severe adenovirus pneumonia, the predominant symptoms include sputum production and dyspnea, with approximately 90% of patients exhibiting fever and over 90% presenting with cough. Notably, fewer than 30% of patients display neurological or gastrointestinal symptoms [8]. This case aligns with these findings, manifesting fever, cough, and sputum at disease onset, alongside white blood cell counts and procalcitonin levels within normal ranges, and a presence of lymphocytopenia, consistent with descriptions in the literature. Adenoviruses exhibit tissue tropism, with their clinical manifestations varying according to the infecting serotype. The HaV‐B21 serotype, initially identified in a patient with conjunctivitis, has been linked to respiratory infections in children. However, cases of respiratory failure and severe ARDS associated with HaV‐B21, particularly the B21a subtype, are relatively rare but have been noted for their severity and potential lethality [9, 10].

For accurate diagnosis of adenovirus pneumonia, etiological evidence is essential. Although virus isolation and serotyping are considered the definitive standard for adenovirus diagnosis, their application in early clinical settings is limited due to the time‐consuming nature of these methods. Currently, the most prevalent clinical diagnostic technique is antigen detection. This method involves analyzing patients' nasopharyngeal secretions, nasopharyngeal swabs, sputum, and bronchoalveolar lavage fluid using the immunofluorescence assay to detect the adenovirus capsid hexon antigen. Notably, the detection rate peaks 3–5 days prior to symptom onset, and in severe cases, the virus may remain detectable for over two weeks [11]. Polymerase chain reaction (PCR) assays offer a quantitative approach to viral detection. Carmen Andrea Pfortmueller highlighted in her 2019 case report the utility of quantifying viral load in alveolar lavage fluid and plasma via PCR to inform clinical management and prognosis assessment [12]. Macrogenome sequencing presents significant benefits for identifying pathogens in patients with critical infections. For infections with non‐specific pathogens, early identification and subsequent targeted therapy are critical to improving patient survival rates.

Currently, the management of severe adenovirus pneumonia lacks specific treatments, with care predominantly supportive. The therapeutic efficacy of pharmacological interventions remains underexplored due to the scarcity of large‐scale, controlled clinical studies. Cidofovir, approved by the US FDA for treating severe adenovirus pneumonia, is not available in China, and its clinical efficacy in this context remains uncertain. A 2019 Swiss study reported the deaths from multiple organ failure of two patients with severe adenovirus pneumonia despite receiving ECMO‐supported cidofovir treatment [12]. While there have been instances of successful outcomes attributed to cidofovir, discerning whether these results stem from the drug's action or other therapeutic influences remains challenging. Ribavirin, a guanosine analog with in vitro efficacy against both DNA and RNA viruses, is indicated for cytomegalovirus infections according to the Chinese pharmacopeia. Jie Gu's 2021 meta‐analysis identified ribavirin as the second most frequently administered medication following cidofovir for adenovirus pneumonia (15/228, 6.6%) [13]. Byung Woo Yoon's 2017 report detailed a successful treatment of adenovirus pneumonia with oral ribavirin in a 39‐year‐old male [14]. Additionally, a 2022 observational study by Shen involving 52 pediatric patients with adenovirus pneumonia found that ribavirin monotherapy significantly shortened the duration of non‐respiratory symptoms [15, 16]. In this case, the patient's oxygenation showed no significant improvement until ribavirin was administered three days post‐admission, at which point a substantial enhancement in oxygenation was observed, highlighting ribavirin's potential effectiveness in this patient.

In the initial phase of severe infections, both pro‐inflammatory and anti‐inflammatory responses are activated concurrently. The dominant response significantly influences the prognosis of critically ill patients. Glucocorticoids serve an essential anti‐inflammatory function in managing severe adenovirus pneumonia by moderating the excessive immune reaction during the infection's early stages and diminishing pulmonary exudation. Concurrently, current guidelines for managing severe ARDS endorse the early application of glucocorticoids and immunoglobulin therapy. In the case discussed, timely administration of glucocorticoid therapy was crucial in reducing pulmonary exudation, and the adoption of a prone position proved pivotal for the obese patient to navigate through the most critical phase on the night of admission.

In conclusion, severe adenovirus pneumonia is associated with a high mortality rate, with treatment predominantly reliant on supportive measures. In scenarios lacking ECMO support, the emphasis on prone position ventilation and ARDS ventilation strategies becomes central to care, while early intervention with glucocorticoids, immunoglobulins, and antiviral therapy serves as complementary approaches.

## Author Contributions

**Ji Peng:** conceptualization, writing – original draft. **Zhen Wang:** funding acquisition, visualization. **Shili Zhong:** conceptualization, writing – original draft, writing – review and editing.

## Consent

Written informed consent was obtained from the patient for publication of this case report and any accompanying images. A copy of the written consent is available for review by the Editor‐in‐Chief of this journal.

## Conflicts of Interest

The authors declare no conflicts of interest.

## Data Availability

The authors have nothing to report.

## References

[1] J. P. Lynch and A. E. Kajon , “Adenovirus: Epidemiology, Global Spread of Novel Serotypes, and Advances in Treatment and Prevention,” Seminars in Respiratory and Critical Care Medicine 37, no. 4 (2016): 586–602, 10.1055/s-0036-1584923. 27486739 PMC7171713

[2] C. Cillóniz , J. M. Pericàs , J. R. Rojas , and A. Torres , “Severe Infections due to Respiratory Viruses,” Seminars in Respiratory and Critical Care Medicine 43, no. 1 (2022): 60–74, 10.1055/s-0041-1740972. 35172359

[3] M. K. Scott , C. Chommanard , X. Lu , et al., “Human Adenovirus Associated With Severe Respiratory Infection, Oregon, USA, 2013–2014,” Emerging Infectious Diseases 22, no. 6 (2016): 1044–1051, 10.3201/eid2206.151898. 27191834 PMC4880082

[4] G. B. Biserni , S. Scarpini , A. Dondi , et al., “Potential Diagnostic and Prognostic Biomarkers for Adenovirus Respiratory Infection in Children and Young Adults,” Viruses 13, no. 9 (2021): 1885, 10.3390/v13091885. 34578465 PMC8472906

[5] G. Saint‐Pierre Contreras , D. Conei Valencia , L. Lizama , D. Vargas Zuñiga , L. F. Avendaño Carvajal , and S. Ampuero Llanos , “An Old Acquaintance: Could Adenoviruses Be Our Next Pandemic Threat?,” Viruses 15, no. 2 (2023): 330, 10.3390/v15020330. 36851544 PMC9966032

[6] D. Kim , E. Lee , J. Eom , et al., “Prevalence and Burden of Human Adenovirus‐Associated Acute Respiratory Illness in the Republic of Korea Military, 2013 to 2022,” Journal of Korean Medical Science 39, no. 4 (2024): e38, 10.3346/jkms.2024.39.e38. 38288539 PMC10825453

[7] J. R. Radke and J. L. Cook , “Human Adenovirus Lung Disease: Outbreaks, Models of Immune‐Response‐Driven Acute Lung Injury and Pandemic Potential,” Current Opinion in Infectious Diseases 36, no. 3 (2023): 164–170, 10.1097/QCO.0000000000000917. 37093048 PMC10133205

[8] T. Lion , “Adenovirus Persistence, Reactivation, and Clinical Management,” FEBS Letters 593, no. 24 (2019): 3571–3582, 10.1002/1873-3468.13694. 31411731

[9] J. S. Sammons , E. H. Graf , S. Townsend , et al., “Outbreak of Adenovirus in a Neonatal Intensive Care Unit: Critical Importance of Equipment Cleaning During Inpatient Ophthalmologic Examinations,” Ophthalmology 126, no. 1 (2019): 137–143, 10.1016/j.ophtha.2018.07.008. 30180976

[10] G. C. Gray , T. McCarthy , M. G. Lebeck , et al., “Genotype Prevalence and Risk Factors for Severe Clinical Adenovirus Infection, United States 2004–2006,” Clinical Infectious Diseases 45, no. 9 (2007): 1120–1131, 10.1086/522188. 17918073 PMC2064001

[11] M. Morozumi , H. Shimizu , Y. Matsushima , et al., “Evaluation of New Immunochromatographic Assay Kit for Adenovirus Detection in Throat Swab: Comparison With Culture and Real‐Time PCR Results,” Journal of Infection and Chemotherapy 20, no. 5 (2014): 303–306, 10.1016/j.jiac.2013.12.003. 24594452

[12] H. S. Huang , C. L. Tsai , J. Chang , et al., “Multiplex PCR System for the Rapid Diagnosis of Respiratory Virus Infection: Systematic Review and Meta‐Analysis,” Clinical Microbiology and Infection 24, no. 10 (2018): 1055–1063, 10.1016/j.cmi.2018.01.005. 29208560 PMC7128951

[13] C. A. Pfortmueller , M. T. Barbani , J. C. Schefold , E. Hage , A. Heim , and S. Zimmerli , “Severe Acute Respiratory Distress Syndrome (ARDS) Induced by Human Adenovirus B21: Report on 2 Cases and Literature Review,” Journal of Critical Care 51 (2019): 99–104, 10.1016/j.jcrc.2019.02.014. 30798099 PMC7172394

[14] J. Gu , Q. Q. Su , T. T. Zuo , and Y. B. Chen , “Adenovirus Diseases: A Systematic Review and Meta‐Analysis of 228 Case Reports,” Infection 49, no. 1 (2021): 1–13, 10.1007/s15010-020-01484-7. 32720128 PMC7962627

[15] B. W. Yoon , Y. G. Song , and S. H. Lee , “Severe Community‐Acquired Adenovirus Pneumonia Treated With Oral Ribavirin: A Case Report,” BMC Research Notes 10, no. 1 (2017): 47, 10.1186/s13104-017-2374-6. 28100279 PMC5241922

[16] K. Shen , Y. Wang , P. Li , and X. Su , “Clinical Features, Treatment and Outcomes of an Outbreak of Type 7 Adenovirus Pneumonia in Centralized Residence Young Adults,” Journal of Clinical Virology 154 (2022): 105244, 10.1016/j.jcv.2022.105244. 35917678

